# Metanephric Adenofibroma Masquerading as Wilms’ Tumor

**DOI:** 10.21699/ajcr.v7i5.463

**Published:** 2016-11-01

**Authors:** Prince Raj, Ashwini Khanolkar, Yogesh Kumar Sarin

**Affiliations:** Department of Pediatric Surgery, Maulana Azad Medical College, New Delhi, India

**Keywords:** Metanephric adenofibroma, Wilms' tumor, Neoadjuvant chemotherapy

## Abstract

Metanephric adenofibroma is a rare, biphasic, benign tumor containing both stromal and epithelial components and could be potentially mistaken as Wilms’ tumor (WT). We present a 5-year-old girl who was suspected to have metastatic Wilms’ tumor on radiological investigations/tru-cut biopsy and had received neoadjuvant chemotherapy, but postoperatively final histopathology revealed it as metanephric adenofibroma. No postoperative chemotherapy was given

## INTRODUCTION

Wilms’ tumor is the most common tumor of kidney in the pediatric age group and is easily diagnosed on biopsy, but with recent recognition of various other pediatric renal mass of different biologic behavior the role of pathologist for correct diagnosis is more important. Metanephric adenofibroma (MAF) is one of the rare benign tumors of kidney that could be easily mistaken for Wilms’ tumor (WT). [1]

## CASE REPORT

A 5-year-old girl presented with progressively increasing abdominal lump for seven months. Lump was not associated with pain, fever, hematuria or vomiting. Examination revealed a large right renal mass involving the right lumbar, right hypochondrium, right iliac fossa and extending into the umbilical and hypogastric regions of the abdomen. Contrast enhanced computed tomography (CECT) abdomen and thorax showed 16cm x13cm x13cm minimally enhancing solid mass lesion involving entire right kidney (Fig.1) and subcentimetric peripheral nodules in left lung. There was no evidence of extension into the adjacent adrenal gland, renal vein, or inferior vena cava. Tru-cut biopsy gave differential diagnoses of mesenchymal variant of Wilms’ tumour and cellular mesoblastic nephroma. Based on findings of clinical, radiological and histopathological examination, provisional diagnosis of metastatic WT was kept. The patient received 6 cycles of AVD regime based chemotherapy considering it to be Stage IV Wilms’ as per SIOP protocol. Post chemotherapy CECT showed complete resolution of lung nodules with no significant change in size of renal mass. Patient underwent right simple nephroureterectomy with lymph node sampling. However, final histopathology report showed tumor composed of bland spindle cells without mitosis and pleomorphism with scattered adenomatous foci composed of small tubules lined by nondescript cuboidal epithelium. These features were suggestive of metanephric adenofibroma with clear margins (Fig.2). In view of the benign nature of the lesion, we decided on no further toxic chemotherapy and monthly follow up with clinical and ultrasound examination to look for any recurrences.Child is doing well on follow up with no evidence of recurrence in last five months.

**Figure:**
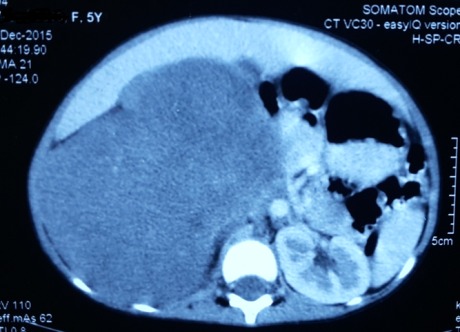
Figure 1:CECT showing right renal mass.

**Figure:**
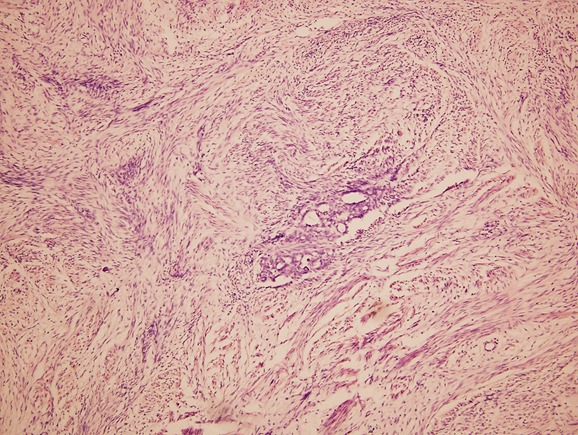
Figure 2:Tumor composed of bland spindle cells without mitosis and pleomorphism with scattered adenomatous foci composed of small tubules lined by nondescript cuboidal epithelium. (H and E x 100).

## DISCUSSION

Hennigar and Beckwith were the first to describe MAF in 1992.[1] It is a rare, biphasic, benign tumor containing both stromal and epithelial components in various proportions. This tumor is classified among the metanephric neoplasms, which also include metanephric adenoma and metanephric stromal tumour. Metanephric adenoma (MA) is a purely epithelial lesion and metanephric stromal tumor (MST) is a purely stromal lesion, while MAF is in between them. MAF lies in a spectrum between the MA and MST and could also merge with the morphology of WT thus it supports the concept that these all are related lesions.[2-4]. In our case too, the tru-cut biopsy was not conclusive of WT as the pathologist suggested a possibility of mesenchymal variant WT and cellular mesoblastic nephroma. Differentiating MAF with cellular mesoblastic nephroma may at times be also difficult, though certain features may be useful in distinguishing this stroma from congenital mesoblastic nephroma (CMN) that includes intratumoral angiodysplasia, concentric cuffing of entrapped tubules ("onion skinning"), and heterologous differentiation.[2] At present no particular immunohistochemistry findings are diagnostic of metanephric adenofibroma. The epithelial component is positive for AE1/3 and WT1, while the stromal component is positive for Vimentin and CD34.[2]

Clinically, the child may present with polycythemia, hypertension, or hematuria, which resolve following nephrectomy.[1] These tumors can achieve significant size and present with a palpable mass in abdomen and it is not possible to distinguish these tumors from other paediatric solid tumors radiologically. As it is a rarity, there is no standard treatment protocol but being a benign tumor simple nephrectomy is curative, though there have been reports of even partial robotic nephrectomy.[5] Majority of patients have good prognosis and don’t have recurrence on follow up.[6] There is no role for chemotherapy or radiotherapy in management of MAF. In our case, neoadjuvant chemotherapy was administered as the tru-cut biopsy diagnosis was WT, though there was no significant reduction in the size of tumor following chemotherapy, but the subcentimetric lung nodules resolved.

Though we cannot conclusively rule out the possibility of malignant metanephric adenosarcoma in view of the subcentimetric lung nodules, but as the histopathology came out be benign we decided to follow up the patient rather than to subject her to further chemotherapy and radiotherapy. Moreover, metanephric adenosarcoma is extremely rare.[7,8] Nevertheless, its possibility has to be kept in mind and a regular follow up is warranted.

To summarize, metanephric adenofibroma, though rare, should be kept as a differential for a large renal masses presenting outside the spectrum of routine Wilms’ tumor presentation. This can avoid toxic chemotherapy and its complications.

## Footnotes

**Source of Support:** Nil

**Conflict of Interest:** None declared

